# A flexible pH sensor based on polyaniline@oily polyurethane/polypropylene spunbonded nonwoven fabric

**DOI:** 10.1039/d3ra07878g

**Published:** 2024-02-13

**Authors:** Xiangxiang Zhu, Hui Sun, Bin Yu, Lei Xu, Hao Xiao, Zhuan Fu, Tian Gao, Xiaodong Yang

**Affiliations:** a College of Textiles Science and Engineering, Zhejiang Sci-Tech University Hangzhou 310018 China sunhui@zstu.edu.cn; b Zhejiang Provincial Innovation Center of Advanced Textile Technology Shaoxing 312000 China; c School of Textile and Clothing and Art and Media, Suzhou Institute of Trade & Commerce 287 Xuefu Road Suzhou 215009 Jiangsu China

## Abstract

To fabricate a two-electrode flexible pH sensor based on polypropylene spunbonded nonwoven fabric (PP SF), oily polyurethane (OPU) was first coated on the surface of PP SF to obtain OPU/PP SF. Then, silver/silver chloride (Ag/AgCl) paste, used as the reference electrode and conductive carbon (C) paste were transferred to the OPU/PP SF surface through screen printing. Polyaniline (PANI) was deposited on the surface of the C paste to form a sensing working electrode *via* the electro-chemical deposition method. The results showed that the surface of the obtained PANI@OPU/PP SF flexible pH sensor (3D PANI pH sensor) presented a three-dimensional (3D) porous network structure. The 3D PANI pH sensor had good mechanical properties, an excellent Nernst response (−67.67 mV pH^−1^) and linearity (*R*^2^ = 0.99) in the pH range from 2.00 to 8.00 in the normal state. In the bent state, the 3D PANI pH sensor retained similar sensitivity (−68.87 mV pH^−1^) and linearity (*R*^2^ = 0.99). Moreover, the 3D PANI pH sensor exhibited a short response time (8 s), excellent reversibility (1.20 mV), low temperature drift (−0.0872 mV pH^−1^ °C^−1^) and long-term stability (0.83 mV h^−1^) in the normal state. Furthermore, the 3D PANI pH sensor can be effectively applied for pH monitoring of liquids and fruits with irregular curved surfaces. The error margin is no more than 0.16 compared to a commercial pH meter.

## Abstract 2

Structure, mechanism, and application of a 3D PANI pH sensor in liquid and fruit pH value detection.

## Graphical abstract

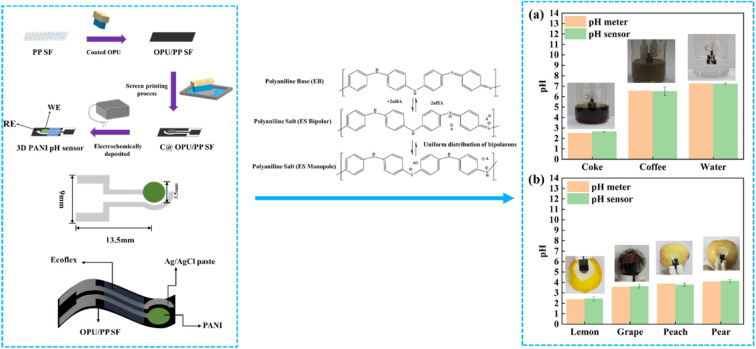

## 1. Introduction

pH is an important monitoring index in environmental, clinical, industrial and food fields.^1–4^ For example, the pH value of fresh fruits and vegetables changes as they gradually decay during the storage process. Research studies have shown that the ammonia secreted by fungi leads to an elevation in the pH of the host tissues of fruits during the fungus infection process.^5^ Therefore, it is necessary to monitor the pH of fruits and vegetables to judge their edibility.^6,7^ The traditional pH meter with a glass electrode can indeed provide reliable detection. However, its limitations in terms of fragility, inflexibility and difficulty in miniaturization have become more and more prominent in actual applications. Thus, the fabrication and application of flexible pH sensors have garnered the attention of researchers.

Generally, a flexible potentiometric pH sensor is composed of a working electrode, a reference electrode, a flexible substrate, and conductive interconnections. These components play crucial roles in controlling the overall functionality and performance of flexible pH sensors. Although not directly participating in sensing, the flexible substrate provides flexibility and strength and protects sensing materials and signals.^8^ Due to the excellent chemical inertness and thermoelectric insulation ability, many researchers regard polymer films as one of the best choices for a flexible pH sensor substrate. Polymer films, such as polyimide (PI),^9^ polydimethylsiloxane (PDMS),^10^ polyethylene terephthalate (PET),^11–14^ Ecoflex^15^ and naphthalene (PEN),^16,17^ have been successfully used to prepare flexible pH sensors. In recent years, the use of fabrics as a substrate for flexible pH sensors has aroused the interest of researchers due to their good mechanical properties, excellent flexibility, and recoverability. Özdemir *et al.*^18^ fabricated a pH-responsive TiO_2_ nanoparticle coating on the surface of cotton fabric *via* the hydrolysis of a TiCl_4_ solution and constructed a potentiometric pH sensor. The flexible pH sensor exhibited a linear potentiometric response in the pH range from 2.0 to 9.0 with a slope of −23.9 mV pH^−1^. Zhao *et al.*^19^ prepared polyaniline (PANI)-modified polyvinylidene fluoride (PVDF) nanofiber yarns using *in situ* polymerization, and these yarns were woven into fabrics to obtain pH-sensitive yarns and fabric sensors, respectively. The yarn sensor exhibited an approximate pH-dependent surface potential of −48.53 mV pH^−1^, while the fabric showed an approximate surface potential of −38.4 mV pH^−1^ within the pH range from 4.0 to 8.0.

However, polymer films and traditional woven and knitted fabrics have complex manufacturing processes and high cost. Nonwoven fabrics have the advantages of a simpler preparation process, cost-effectiveness, and high yield, and hence are often used as disposable products.^20^ Polypropylene spunbonded nonwoven fabric (PP SF) is one of the most common nonwoven fabrics due to its excellent chemical stability, good mechanical properties and low cost and has been widely used in medical products, industrial applications, and agricultural applications. However, there are only few reports on flexible pH sensors based on nonwoven fabric substrates.^21^

The working electrode is responsible for interacting with the analyte and converting the pH signal into a measurable electrical signal. Conductive polymers with pH-sensitivity can be used as sensing materials for flexible pH sensors.^22,23^ Among them, PANI is one of the most popular pH-sensitive materials because it exhibits multiple oxidation states and the reversible protonation and deprotonation conversions of emeraldine base (EB) and emeraldine salt (EA) moieties. Some sensing performance parameters of flexible pH sensors using PANI as a sensitive material are shown in Table 1. PANI is easily synthesized, and its structure and performance can be controlled by the adjustment of synthesis conditions and methods. Due to its higher surface-to-volume ratio and capacity of accommodating a greater number of H^+^ binding sites, PANI possesses the enhanced pH-responsive properties.^24–26^

**Table 1:** Structures and sensing performance of different pH sensors

Substrate	Material	pH range	Response time(s)	Sensitivity (mV pH^−1^)	Hysteresis (mV)	Drift (mV h^−1^)	Reference
PET	PANI	3.9–10.1	12.8	−62.4	5.6	3.0 (pH 5.5)	6
PANI/PVDF fabric	PANI	4.0–8.0	30	−38.4	—	—	19
—	3D PANI	4–9	7.75	−69.33	3.8	0.1 (pH 7)	24
PET	PANI	2.38–11.61	<1	∼−60.3	1.9	0.64 (pH 5)	27
						0.49 (pH 7)	
PEDOT-MWCNT-cotton yarns	PANI	2.0–12.0	—	−61 ± 2	—	—	28
Waterproof polyester	PANI, CNTs	5–9	—	−45.9	1	—	29
PP SF	3D PANI	2–8	8	−67.67	1.20	0.83 (pH 7)	This work

The reference electrodes can provide a stable reference potential for accurate pH measurements. Ag/AgCl is often used as the reference electrode in view of its potential stability and environmental friendliness. Yoon *et al.*^27^ fabricated flexible pH sensors using a two-electrode configuration composed of a PANI nanopillar array working electrode and an Ag/AgCl reference electrode. This pH sensor demonstrated a near-Nernstian response with a sensitivity of −60.3 mV pH^−1^ within the pH range from 2.38 to 11.61. Park *et al.*^6^ developed a flexible pH sensor including a PANI-based sensitive electrode and an Ag/AgCl reference electrode. This flexible pH sensor had a high sensitivity of −62.4 mV pH^−1^ within the pH range from 3.9 to 10.1.

In this study, PP SF was chosen as the substrate, oily polyurethane (OPU) was first coated on the surface of PP SF in consideration of the porosity and rough surface of PP SF, and OPU/PP SF was obtained. Then, PANI was deposited on the surface of OPU/PP SF as a working electrode, and Ag/AgCl was used as the reference electrode to prepare the PANI@OPU/PP SF flexible pH sensor (3D PANI pH sensor). The 3D PANI pH sensor was characterized in terms of surface morphology, chemical analysis, mechanical properties, and sensing properties. It is hoped that our study might serve as a reference for the research of flexible pH sensors based on nonwoven fabric substrates.

## 2. Experimental

### 2.1. Materials

PP SF was obtained from Hangzhou Elsite Industrial Co., Ltd (its areal density is about 71 g m^−2^). Aniline (99.5%), phytic acid (50%), polyvinyl butyraldehyde (PVB), citric acid, methanol, disodium hydrogen phosphate dodecahydrate (Na_2_HPO_4_·12H_2_O), potassium chloride (KCl), calcium chloride (CaCl_2_), magnesium chloride (MgCl_2_), and ammonium chloride (NH_4_Cl) were obtained from Macklin Biochemical Co., Ltd. (Shanghai, China). Sulfuric acid (H_2_SO_4_), sodium chloride (NaCl), hydrochloric acid and sodium hydroxide (NaOH) were purchased from Aladdin Industrial Corporation (Shanghai, China). Silver/silver chloride (Ag/AgCl) paste was bought from Shanghai Julong Electronic-technology Co., Ltd. Ecoflex was purchased from Smooth-On (U.S.A). Carbon conductive ink was purchased from Jiewei Screen Printing Co., Ltd (Shenzhen, China). OPU was purchased from Haobo Waterproof Material Co., Ltd. All materials were used as received without any further purification. The screen-printing mask was customized by Dabaicai Screen Printing Co., Ltd. Deionized water (DI) was employed in all experiments.

### 2.2. Fabrication of the 3D PANI pH sensor

**Fig. 1:**
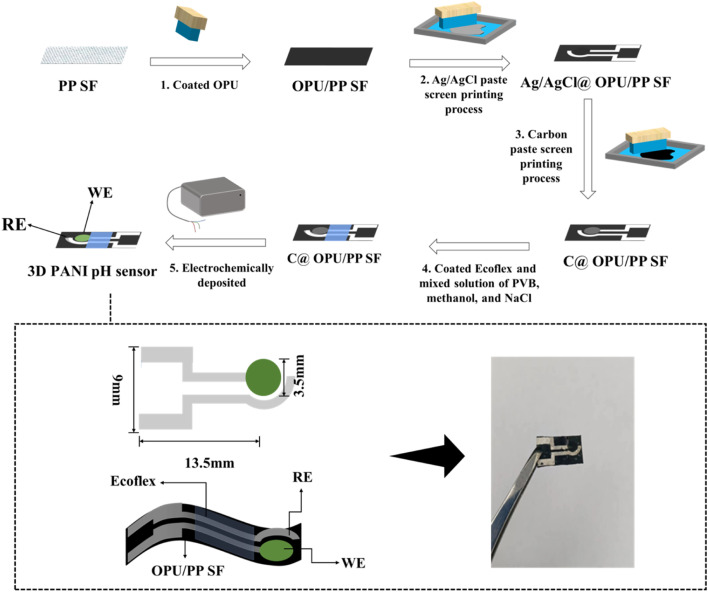
Fabrication route of the 3D PANI pH sensor.

Prior to the experiment, PP SF was scissored into 10 × 18 mm^2^. OPU was coated on the surface of PP SF and dried for 4 h at 60 °C to obtain OPU/PP SF. Subsequently, the Ag/AgCl paste was transferred onto the surface of OPU/PP SF by use of the screen-printing mask and dried at 60 °C for 20 min, and Ag/AgCl@OPU/PP SF was obtained. After that, the conductive carbon ink with a diameter of 3.5 mm, as the C electrode, was transferred onto the surface of the Ag/AgCl electrode by screen printing and dried at 60 °C for 7 min to prepared C@OPU/PP SF. Then, 50 mg of NaCl, 78 mg of PVB, and 1 mL of methanol were mixed and put on the surface of the Ag/AgCl electrode and dried at 60 °C for 9 min. Ecoflex was formed on the electrode surface as an encapsulation layer at 60 °C for 9 min. After that, PANI was prepared on the surface of the C electrode by the cyclic voltammetry technique. The electrode was placed in a three-electrode system and electrochemically deposited for 10 cycles in the potential range from −0.2 to 1.0 V. The electrolyte was a mixture of 0.18 mL of aniline monomer, 0.33 mL of phytic acid and 18 mL of 1 M H_2_SO_4_ solution. The sample was taken out and washed five times with DI and dried at room temperature for 24 h. Finally, the 3D PANI pH sensor was obtained. Fig. 1 is the fabrication route of the 3D PANI pH sensor.

### 2.3. Physical characterization and electrochemical measurements

The surface morphologies of samples were analyzed using scanning electron microscopy (SEM, Zeiss Compact) at an accelerating voltage of 3 kV. Prior to SEM analysis, each sample was gold-plated for 100 seconds. The spectroscopy properties of the samples were characterized by infrared spectroscopy (FT-IR, Nicolet 57000) in the range of 4000–500 cm^−1^. These measurements were performed in attenuated total reflection (ATR) mode, and thirty-two scans were conducted per sample. Electrochemical deposition and electrochemical performance testing were carried out on a CHI660e electrochemical analyzer manufactured by Shanghai Chenhua Company. The pH buffer solution used during the measurements was prepared by mixing 0.1 M of citric acid with 0.2 M of disodium hydrogen phosphate in various ratios. In addition, the pH level was confirmed by a commercial pH meter (PHS-3C) from Shanghai INESA Scientific Instrument Co., Ltd. A universal tensile testing machine (Instron 3369S3163) was used to evaluate the mechanical properties of the samples, where the tensile speed was 50 mm min^−1^, and the length of the gauge was 20 mm. Each sample was tested five times.

**Fig. 2:**
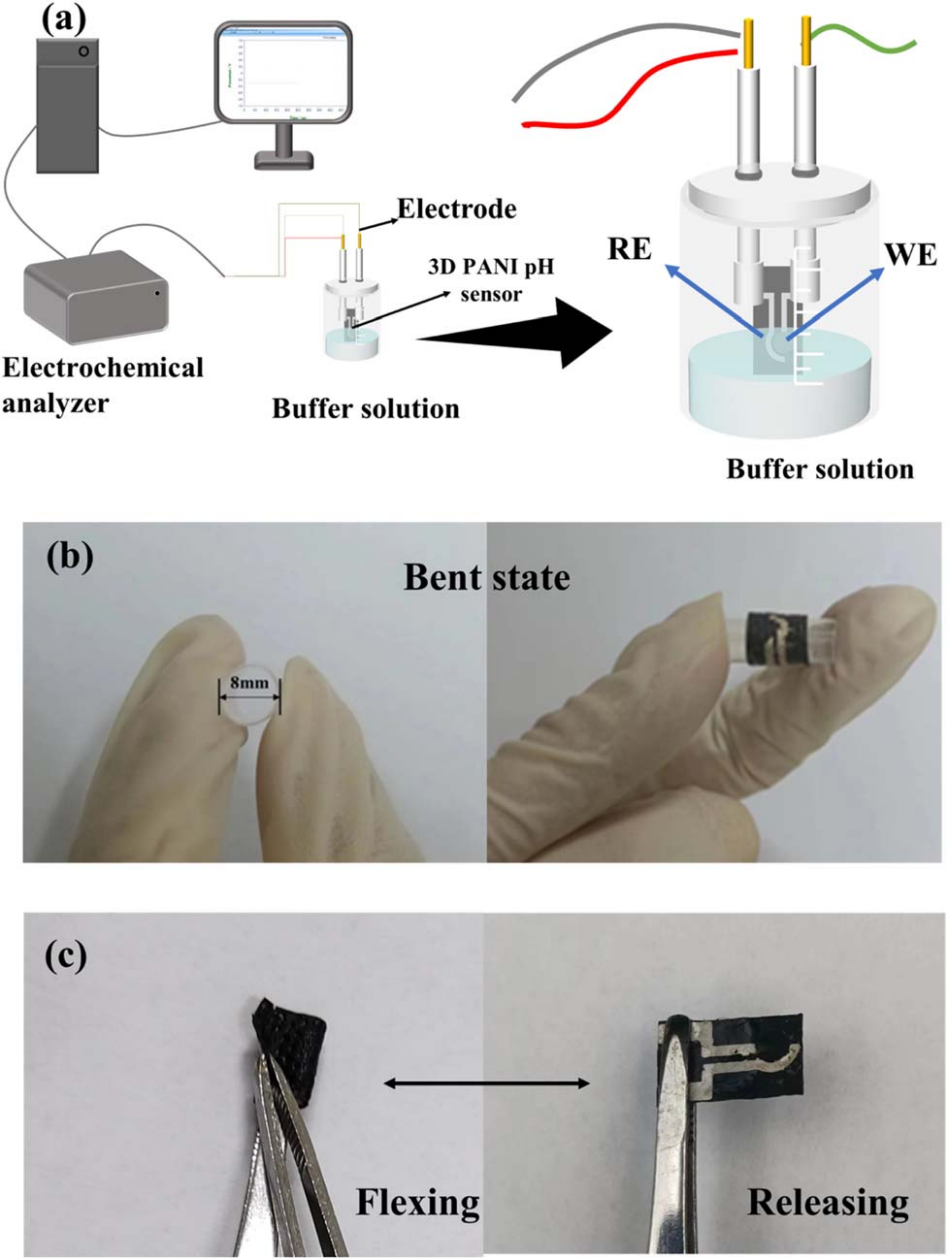
A diagram of the device used for testing the sensing performance of the 3D PANI pH sensor.

Fig. 2(a) depicts a schematic of the device used for the sensing performance measurement of the 3D PANI pH sensor. The electrochemical workstation was connected to a 3D PANI pH sensor in the buffer solution for testing. OCP (open circuit potential) obtained through this measurement served as the primary measurement signal for this work. In the normal state, the OCP signal was recorded when the 3D PANI pH sensor reached a stable signal in buffer solutions with pH values of 2.00, 3.00, 4.00, 5.00, 6.00, 7.00, and 8.00, respectively. The OCP–time curves and OCP–pH curves were plotted.

The sensing performance of the 3D PANI pH sensor in the bent state was tested according to Fig. 2(b). The 3D PANI pH sensor was fixed on a hard plastic pipe with a bent diameter of 8 mm for measurement.

Fig. 2(c) depicts the flexing–releasing cyclic testing mechanism of the 3D PANI pH sensor. The number of bends is a challenge to the sensing performance of the flexible pH sensor. Our 3D PANI pH sensor was bent up to 1000 times. A test was conducted after every two hundred flexing–releasing cycles.

The response time of the 3D PANI pH sensor was evaluated based on the plot of OCP variation *vs.* time in the normal state from pH = 4.10 to pH = 5.07. The electrochemical potential of the flexible pH sensor will change with working conditions, which is called the memory effect or hysteresis.^30^ The value of the potential change can be used to measure the reversibility of the sensor. In this study, our 3D PANI pH sensor was immersed in a buffer solution of pH = 2.19, 4.10, 6.01, and 7.50, and a reciprocating cycling test was carried out in normal and bent states, respectively. The 3D PANI pH sensor in the normal state was tested in a buffer solution with a pH value of 7.00 for 21 h to measure the drift according to the previous method. The OCP change from 5 to 12 h was regarded as the judgment of the drift rate (mV h^−1^) of the sensor.^27^ Additionally, the 3D PANI pH sensor in the normal state was subjected to a sensing performance test in buffered solutions with pH values from 2.00 to 8.00 at the temperatures of 15 °C, 20 °C, 25 °C, 30 °C, 35 °C, and 40 °C.

Furthermore, five 3D PANI pH sensors were prepared using the same fabrication process as described in Section 2.2 and tested in buffered solutions with pH values from 2.00 to 8.00 in normal and bent states to assess the stability of pH sensor fabrication.

The difference between anodic and cathodic currents of the double-layer is equal to twice the product of the scan rate (*v*) and electrochemical double-layer capacitance (*C*_DL_), as given by eqn (1):^31^

1 Δ*J* = *C*_DL_ × 2 × *v*,

where Δ*J* is the difference between anodic and cathodic currents and *v* is the scan rate. Therefore, the slope of the current density-scan rate curve represents *C*_DL_. In addition, the electrochemical active surface area (ECSA) of the electrode sample is calculated from the double layer capacitance according to eqn (2):2
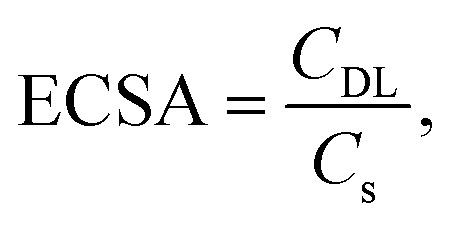
where *C*_s_ represents the specific capacitance of the sample under identical electrolyte conditions or the unit area capacitance of the atomic smooth planes of the material.^32^

In our measurements, the cyclic voltammetry technique was used in a 0.1 M KCl solution, and the scan rate was set from 10 mV s^−1^ to 100 mV s^−1^. The cyclic voltammograms collected from the non-faradaic region were analyzed to calculate the ECSA before and after electrode modification.

The testing accuracy of the flexible pH sensor will be affected by various interfering ions when it is actually used. Hence, it is imperative to assess the ion selectivity of the 3D PANI pH sensor. The separation solution method (SSM) is recommended by the IUPAC to test the ion selectivity of the pH sensor for Ca^2+^, Mg^2+^, K^+^, Na^+^ and NH_4_^+^. The selectivity coefficient of plasma can be obtained from the potential response in different cationic solutions with the same concentration (the concentration of the cationic solution is 0.01 M in this study). The ion selectivity coefficient (*K*) can be expressed as follows:^33,34^3
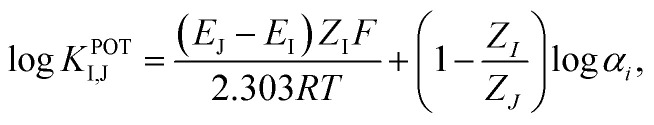
where I is the primary ion, J is the interfering ion, and POT is the potential. *R* is the gas constant, *T* is the temperature, and *F* is the Faraday constant.

### 2.4. Application

Our 3D PANI pH sensor was used to monitor the pH values of three liquids (coke, coffee and water) and four fruits (lemon, grape, peach, and pear) bought from a local supermarket. The 3D PANI pH sensor was placed into each of the liquids for 5 min for testing. The surfaces of the four fruits were first cut to expose the fruit pulp. Then, the 3D PANI pH sensor was affixed onto the exposed surface of the fruits for detection. Five replicates were conducted for each sample. Additionally, the pH values of all liquids and fruits were tested by use of a commercially available pH meter to compare with the 3D PANI pH sensor. The four fruits needed to be squeezed to release juice before being tested by the commercial pH meter.

## 3. Results and discussion

### 3.1. Surface morphology of the flexible pH sensor

**Fig. 3:**
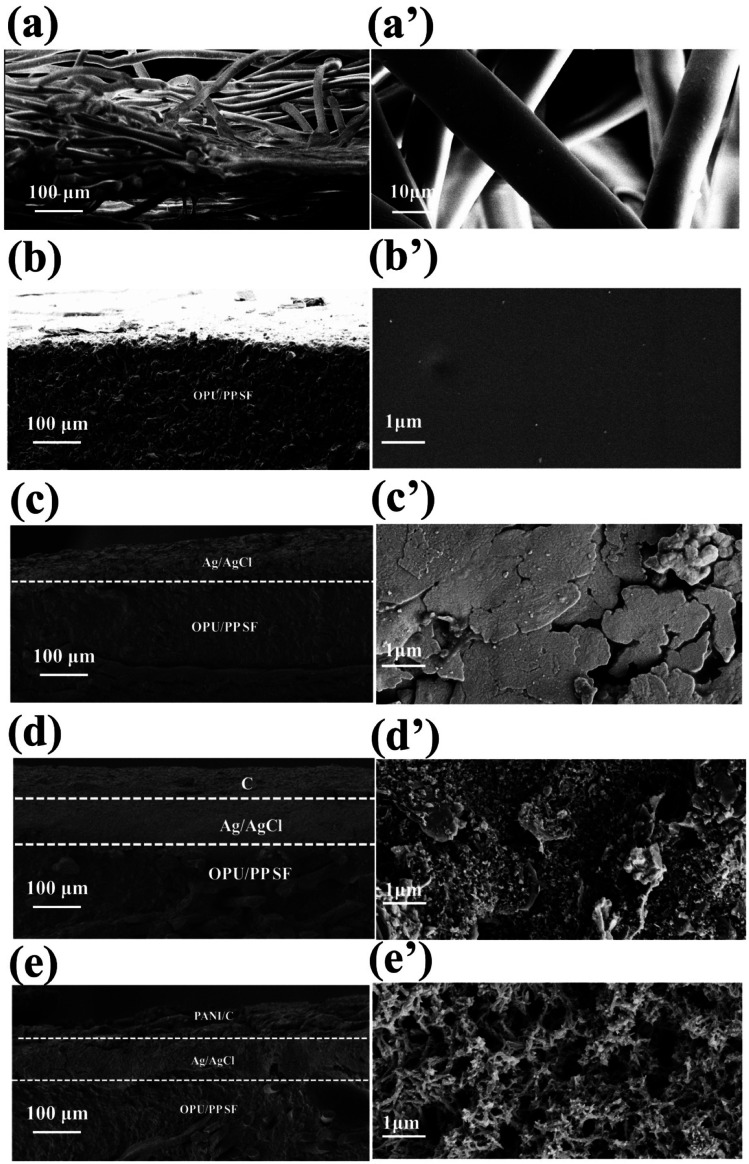
SEM images of the (a–e) cross-section and (a′–e′) surface of PP SF, OPU/PP SF, Ag/AgCl@OPU/PP SF, C@OPU/PP SF, and 3D PANI pH sensors.

Fig. 3 shows cross-sectional and surface SEM images of PP SF, OPU/PP SF, Ag/AgCl@OPU/PP SF, C@OPU/PP SF, and 3D PANI pH sensors. In the cross-sectional image of PP SF, PP fibers are intersected and randomly arranged (Fig. 3(a)), and the fiber surface of PP SF is smooth (Fig. 3(a′)). From the cross-sectional image of OPU/PP SF (Fig. 3(b)), it can be seen that the PP SF is covered and fixed by OPU, and Fig. 3(b′) shows that the surface of OPU/PP SF is smooth and flat compared with PP SF. Fig. 3(c) and (c′) show that after the Ag/AgCl screen is printed, a dense layer of Ag/AgCl with a sheet-like structure is formed on the surface of OPU/PP SF. In Fig. 3(d), a carbon layer is coated on the surface of Ag/AgCl@OPU/PP SF. Fig. 3(d′) shows that the surface of C@OPU/PP SF become rough compared with Ag/AgCl@OPU/PP SF. After PANI is deposited onto the surface of C@OPU/PP SF, it can be seen that the PANI layer is rugged (Fig. 3(e)), and a 3D PANI porous network structure is formed (Fig. 3(e′)). This is attributed to the incorporation of phytic acid, which promotes the interlinking of PANI chains.

### 3.2. Chemical structures

**Fig. 4:**
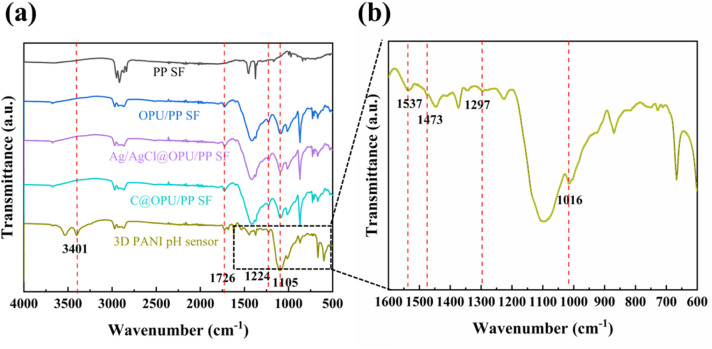
(a) FT-IR spectra of PP SF, OPU/PP SF, Ag/AgCl@OPU/PP SF, C@OPU/PP SF and 3D PANI pH sensors, and (b) magnified FT-IR spectrum with the wavenumber ranging from 1600 to 600 cm^−1^.

FT-IR spectra of PP SF, OPU/PP SF, Ag/AgCl@OPU/PP SF, C@OPU/PP SF and 3D PANI pH sensor samples are shown in Fig. 4(a). In the FTIR spectrum of PP SF, the peaks at 2948 cm^−1^, 2919 cm^−1^, 2865 cm^−1^, 2837 cm^−1^, 1456 cm^−1^ and 1375 cm^−1^ can be assigned to the characteristic peaks of C–C and C–H groups of PP.^35^ Compared to PP SF, the OPU/PP SF spectrum shows C=O stretching at 1726 cm^−1^, the extension of polyester C–O at 1224 cm^−1^, and C–O–C stretching at 1105 cm^−1^.^36,37^ The FT-IR spectra of Ag/AgCl@OPU/PP SF and C@OPU/PP SF do not exhibit new peaks compared with OPU/PP SF. The FT-IR spectrum of the 3D PANI pH sensor shows the –OH of phytic acid at 3401 cm^−1^. The peaks at 1537 cm^−1^ and 1473 cm^−1^ correspond to the C=C stretching of the quinone ring and the benzene ring of PANI, respectively. The peak at 1297 cm^−1^ is attributed to the C–N of the benzene ring unit stretching vibration, and the peak at 1016 cm^−1^ is for C=N stretching.^27^ It is evident from FT-IR data that the 3D PANI pH sensor is successfully fabricated.

### 3.3 Mechanical properties of the flexible pH sensor

**Fig. 5:**
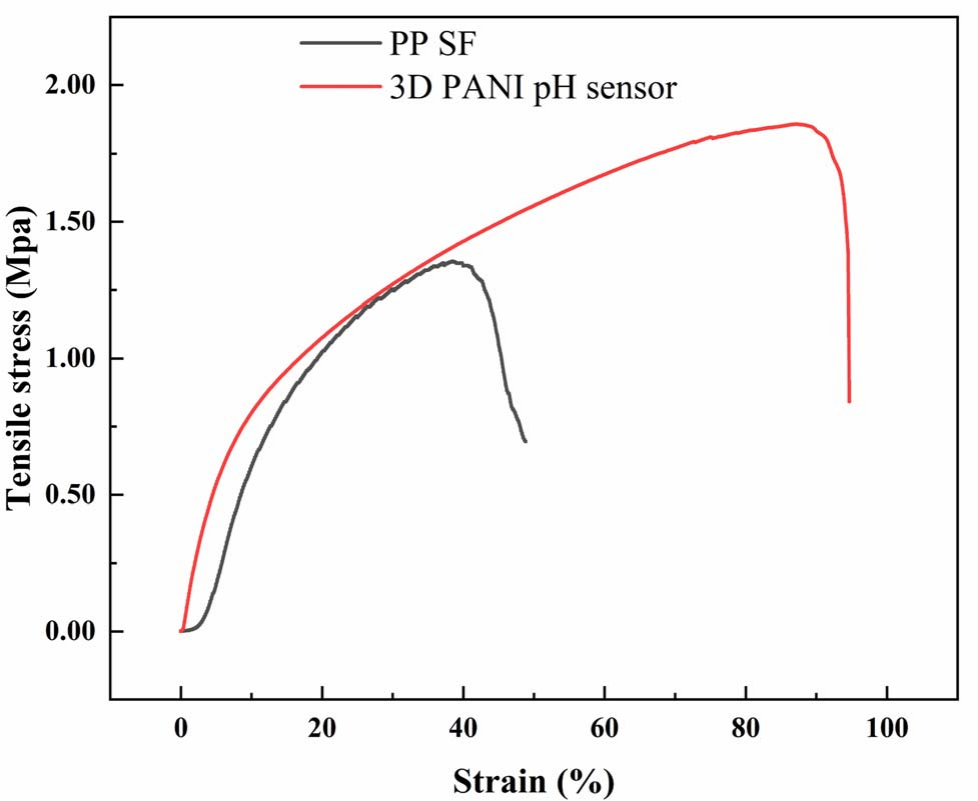
Stress–strain curves of PP SF and the 3D PANI pH sensor.

Fig. 5 shows the stress–strain curves of PP SF and 3D PANI pH sensors. It is seen that the elongation at the break of PP SF is about 38.50%, and the tensile strength is about 1.36 MPa. The 3D PANI pH sensor exhibits an elongation at the break of about 87.50%, representing an improvement of 127.27% compared to the initial PP SF. The tensile strength of the 3D PANI pH sensor is about 1.85 MPa, which is an enhancement of 36.03%. The improvement in mechanical properties of the 3D PANI pH sensor can be attributed to the application of the OPU coating. When OPU penetrates between PP SF fibers, the mobility of PP fibers is limited, which reinforces the whole fiber web.^38^ The excellent mechanical properties of the 3D PANI pH sensor may provide a wider field of application and longer operational lifetimes.

### 3.4. pH sensing performances of the 3D PANI pH sensor

**Fig. 6:**
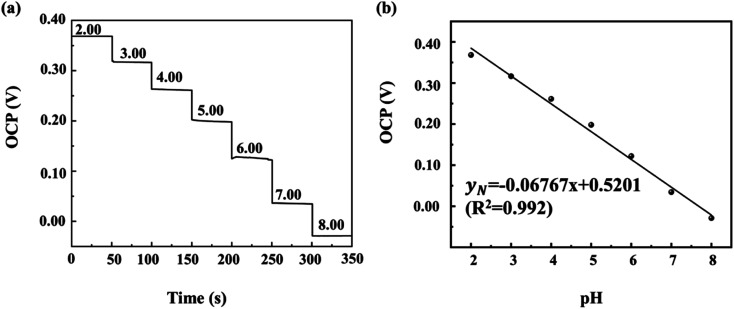
(a) OCP–time curves and (b) the fitted standard calibration line of OCP–pH of 3D PANI pH sensors in the normal state.

Fig. 6 depicts OCP–time curves and the fitted standard calibration lines of OCP–pH curves of the 3D PANI pH sensor in the normal state. The resultant calibration curve is well linear (*R*^2^ = 0.99) with a slope of −67.67 mV pH^−1^ in a wide pH range from 2.00 to 8.00 in the normal state. Its sensitivity is superior to other flexible pH sensors based on the fabric mentioned in Table 1.

**Fig. 7:**
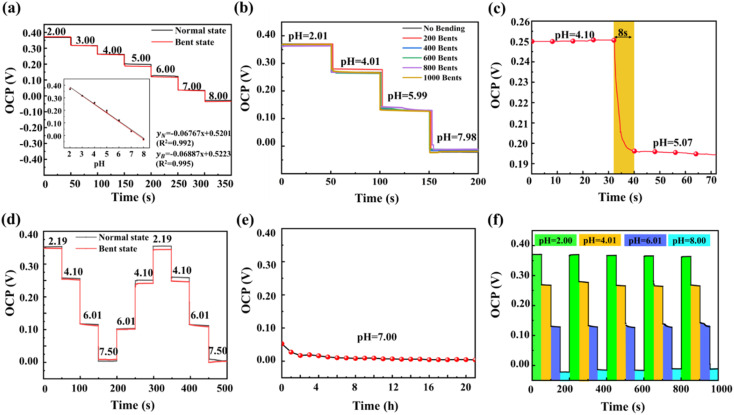
OCP–time curves of 3D PANI pH sensors (a) in buffer solution with the inset showing the fitted standard calibration line in normal and bent states, (b) in buffer solutions with different pH values after 1000 testing cycles of flexing–releasing, (c) in buffer solution from pH 4.10 to 5.07 in the normal state, and (d) after reversibility testing in normal and bent states in buffer solutions with different pH values; (e) OCP–time curves of 3D PANI pH sensors for 21 h in pH 7.00 buffer solution in the normal state and (f) in the normal state after five testing cycles in buffer solutions with different pH values.

Fig. 7(a) shows the OCP–time curves of the 3D PANI pH sensor and the fitted standard calibration line in normal and bent states. The sensitivity (−68.87 mV pH^−1^) and linearity (*R*^2^ = 0.99) of the 3D PANI pH sensor in the bent state remain is similar to those in the normal state, indicating good sensitivity regardless of the states.

Fig. 7(b) depicts the OCP–time curves of the 3D PANI pH sensor measured in buffer solutions with different pH values after 1000 testing cycles of flexing–releasing. The results demonstrate that an average sensitivity of −64.41 mV pH^−1^ is obtained from six separate trials, and the relative standard deviation (RSD) is 1.69%, which is attributed to the good flexibility and response performance of the 3D PANI pH sensor.^24^

Fig. 7(c) shows that when the pH of the buffer solution changes from 4.10 to 5.07, 90% of the equilibrium potential value of the 3D PANI pH sensor is achieved in just 8 s in the normal state, which means a faster response time of our flexible pH sensor than those of PANI flexible pH sensors listed in Table 1.^6,19^Fig. 7(d) shows the reversibility testing of the 3D PANI pH sensor in buffer solutions with different pH in normal and bent states. In the initial normal state, the OCP of the 3D PANI pH sensor is 353.60 mV in the buffer solution with a pH of 2.19. After two cycles, the hysteresis width is only 1.20 mV, which is lower than the same type of flexible pH sensor listed in Table 1.^6,24,27^ Conversely, in the bent state, the hysteresis width of the 3D PANI pH sensor is only 5.60 mV after two cycles. Therefore, it is suggested that the 3D PANI pH sensor has good reversibility.

Fig. 7(e) shows the low drift rate of 0.83 mV h^−1^ when the 3D PANI pH sensor is observed from 5 h to 12 h at pH = 7.00 in the normal state, which is lower than the PANI flexible pH sensor prepared by Park *et al.*,^6^ indicating that the 3D PANI pH sensor has excellent long-term potential stability.

Fig. 7(f) depicts the OCP–time curve of the 3D PANI pH sensor after five testing cycles in buffer solutions with different pH values in the normal state. The results demonstrate that the RSD of the 3D PANI pH sensor is just 1.53%, indicating the remarkable repeatability during continuous operation.

**Fig. 8:**
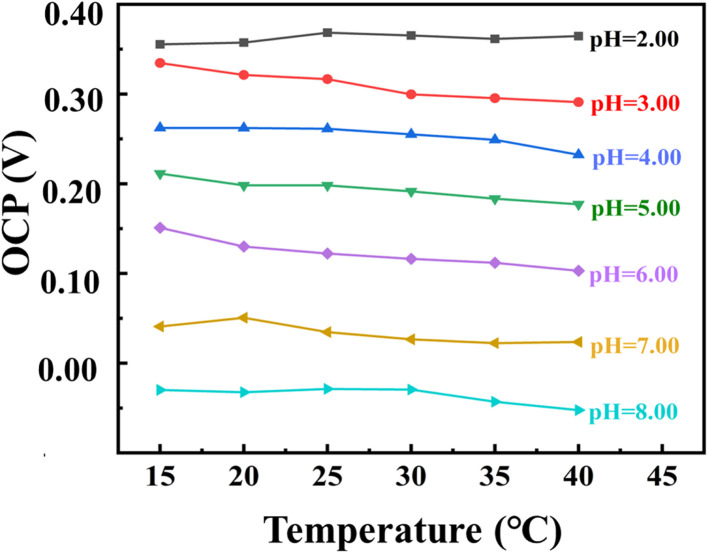
OCP–temperature curves of 3D PANI pH sensors in buffer solutions with the pH changing from 2.00 to 8.00 in the normal state.

Fig. 8 illustrates the OCP–temperature curves of the 3D PANI pH sensor in buffer solutions with different pH values in the normal state when the temperature changes from 15 °C to 40 °C. The results show that the 3D PANI pH sensor exhibits a sensitivity of −66.23 mV pH^−1^ at 15 °C and −68.41 mV pH^−1^ at 40 °C. The temperature drift is just −0.0872 mV pH^−1^ °C^−1^, which guarantees the testing accuracy of our 3D PANI pH sensor when it is applied at different temperatures.

**Fig. 9:**
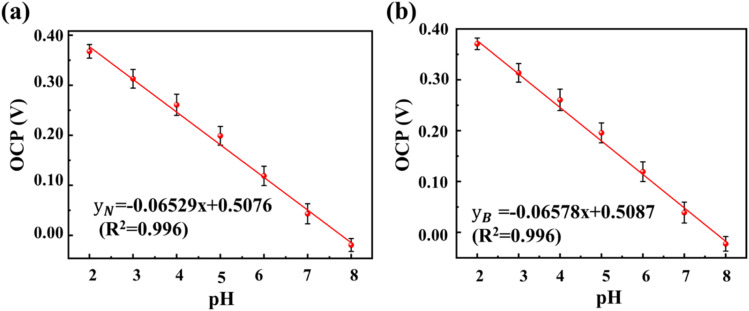
Fitted OCP–pH curves of 3D PANI sensors in the (a) normal state and (b) bent state in pH 2.00–8.00 buffer solutions during the reproducibility test.

Fig. 9 depicts the OCP–pH curves of five 3D PANI pH sensors prepared in buffer solutions with the pH changing from 2.00 to 8.00 in the normal and bent states. The results indicate that the standard deviation (SD) of the sensitivity for the prepared five 3D PANI pH sensors is 0.16% in the normal state and 0.12% in the bent state, demonstrating very low uncertainty in the fabrication process of 3D PANI pH sensors.

**Fig. 10:**
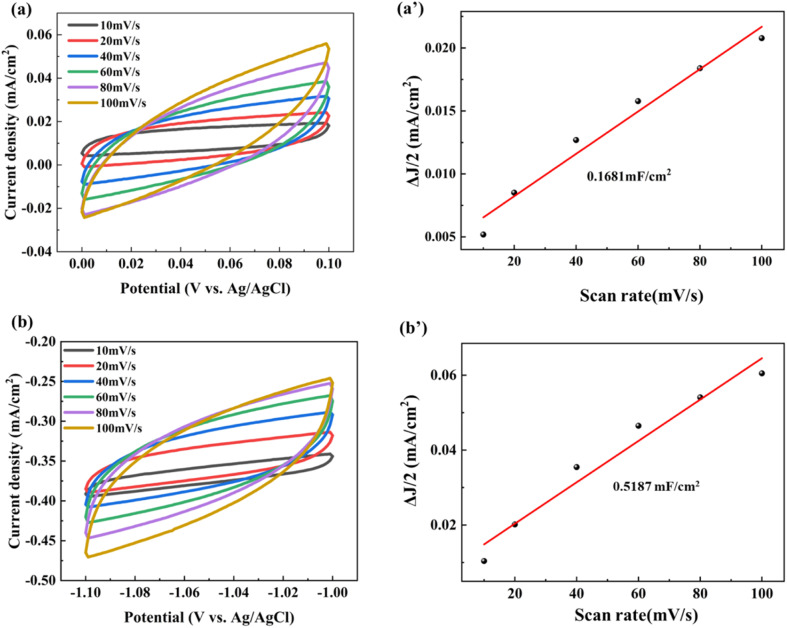
Cyclic voltammetry curves for (a) the C electrode and (b) 3D PANI electrode, and corresponding Δ*J*/2–scan rate fitting curves for (a′) the C electrode and (b′) 3D PANI electrode.

Fig. 10 illustrates the cyclic voltammetry curves of the unmodified C electrode of C@OPU/PP SF and 3D PANI electrode of the 3D PANI pH sensor under different scan rates, and *C*_DL_ is calculated from cyclic voltammetry results. The results indicate that the *C*_DL_ of the unmodified C electrode is 0.1681 mF cm^−2^, and the ECSA is 4.21 cm^2^. Meanwhile, the *C*_DL_ of the 3D PANI electrode is 0.5187 mF cm^−2^, and the ECSA is 12.97 cm^2^. Obviously, the ECSA of the 3D PANI electrode is triple that of the unmodified C electrode. The higher ECSA enhances the space and channel dimensions for ion adsorption, thereby increases the electrochemical activity of the 3D PANI electrode and results in the faster charge propagation behavior for the prepared 3D PANI.^24^

Table 2 depicts the selectivity coefficients of the 3D PANI pH sensor. All *K* values of our 3D PANI sensor are below 10^−4^. IUPAC pointed out that if the *K* value is less than 1, the pH sensor can accurately measure the H^+^ in interfering ions.^6^ Therefore, it is believed that our 3D PANI pH sensor has the impressive ion selectivity.

**Table 2:** Selectivity coefficients of the 3D PANI pH sensor using SSM for the primary ion (H^+^) against interfering ions

Ion (J)	log *K*^POT^_I,J_	*K* ^POT^_I,J_
K^+^	−4.42	3.80 × 10^−5^
Na^+^	−4.15	7.08 × 10^−5^
Ca^2+^	−5.16	6.92 × 10^−6^
Mg^2+^	−5.49	3.24 × 10^−6^
NH_4_^+^	−4.20	6.31 × 10^−5^

### 3.5. Sensing mechanism of the 3D PANI pH sensor

**Fig. 11:**
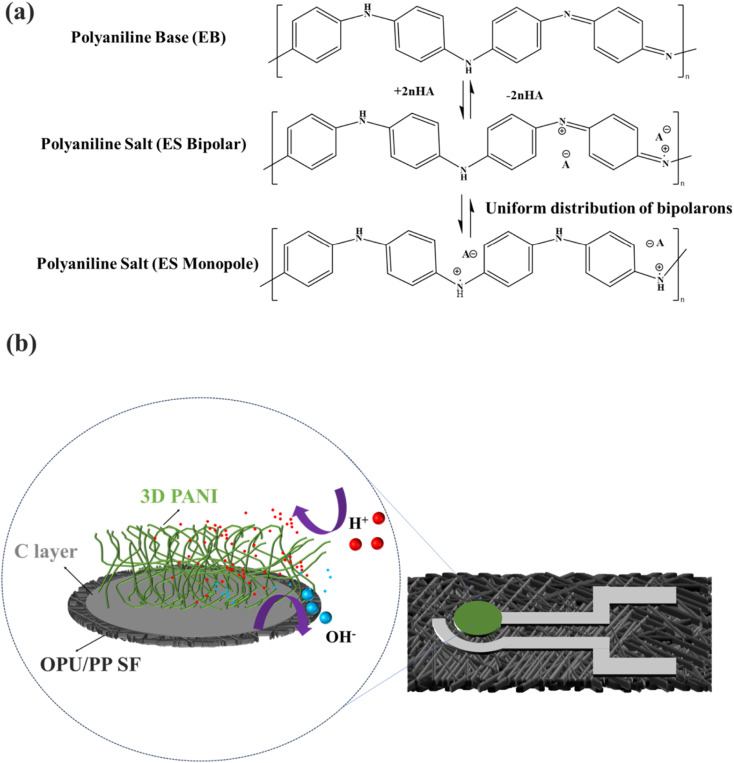
Scheme of the protonation-deprotonation (a) mechanism and (b) pathway for 3D PANI.

Fig. 11(a) displays the sensing mechanism of the 3D PANI pH sensor.^9,24^ In an acidic solution, PANI is doped with H^+^ and generates highly conductive ES, resulting in a decrease in the surface resistance of the 3D PANI pH sensor and a change of the voltage. However, in an alkaline solution, the H^+^ captured by PANI is neutralized by OH^−^, which causes an increase in resistance and generates the opposite effect of the voltage compared to acidic conditions.^9,24^ To evaluate the sensitivity of the 3D PANI pH sensor, the Nernst equation is introduced as follows:4
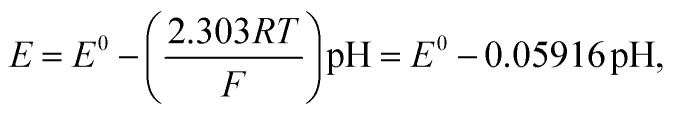
where *E*^0^ is the standard electrode potential, *R* is the gas constant, *T* is the absolute temperature in K, and *F* is the Faraday constant (9.64 × 10^4^ C mol^−1^). Theoretically, the maximum sensitivity of a pH sensor based on the Nernst behavior at room temperature (298 K) is −59.16 mV pH^−1^.^6^ The 3D PANI pH sensor has higher sensitivity (−67.67 mV pH^−1^) because of the formation of PANI with a 3D porous network structure on the surface.

Fig. 11(b) delineates the structural advantages of 3D PANI pH sensor in the protonation-deprotonation mechanism and pathway. The 3D PANI structure and large ECSA (12.97 cm^2^) effectively enhance interaction opportunities with H^+^. Moreover, compared to particulate PANI, PANI with a 3D porous network structure can exhibit higher sensitivity and stronger conductivity.^24^

### 3.6. Application

**Fig. 12:**
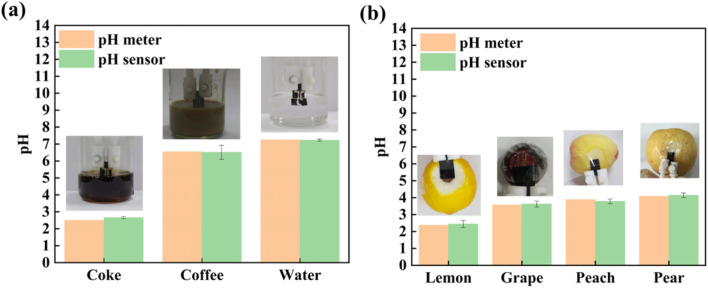
Practical application of the 3D PANI pH sensor for monitoring the pH of (a) three kinds of liquids (coke, coffee and water) and (b) four kinds of fruits (lemon, grape, peach, and pear).

Fig. 12 shows the practical application of the 3D PANI pH sensor in monitoring the pH of three liquid (coke, coffee and water) and four fruits (lemon, grape, peach, and pear). It can be seen that when the prepared 3D PANI pH sensor is used, the pH values of coke, coffee and water are 2.66, 6.51 and 7.23, respectively. Additionally, their pH values tested by the commercially available pH meter are 2.50, 6.55 and 7.25, respectively (Fig. 12(a)). Compared with the commercially available pH meter, the error precision of our 3D PANI pH sensor for three liquids is no more than 0.16. Moreover, when the 3D PANI pH sensor is used, the tested pH values of lemon, grape, peach, and pear were 2.44, 3.62, 3.78 and 4.16, respectively. After squeezing the juice of the four fruits, their pH values tested by the commercially available pH meter were 2.37, 3.57, 3.88 and 4.08, respectively (Fig. 12(b)). Compared with the commercially available pH meter, the error precision of the 3D PANI pH sensor for four fruits is no more than 0.10. The practical application results prove that the prepared 3D PANI pH sensor can be used to monitor the pH values of common liquids and fruits.

## 4. Conclusion

To develop the 3D PANI pH sensor, OPU was first coated onto the surface of PP SF. Subsequently, Ag/AgCl and C were transferred onto the surface of OPU/PP SF through screen printing. Finally, PANI with a 3D porous network structure was electrodeposited as the working electrode, and a 3D PANI pH sensor was obtained. The 3D PANI pH sensor shows good mechanical properties, sensitivity (−67.67 mV pH^−1^) and linearity (*R*^2^ = 0.99) in the pH range from 2.00 to 8.00 in the normal state. In the bent state, the 3D PANI pH sensor has the similar sensitivity and linearity to those in the normal state. After up to 1000 cycles of flexing–releasing, the RSD of the sensitivity of the 3D PANI pH sensor was just 1.69%. Meanwhile, the 3D PANI pH sensor had a short response time (8 s) and long-term stability of 0.83 mV h^−1^ in the normal state, a low potential hysteresis (1.20 mV in the normal state and 5.60 mV in the bent state), a low temperature drift (−0.0872 mV pH^−1^ °C^−1^), excellent repeatability (RSD of sensitivity of 1.53%), and the impressive ion selectivity. The 3D PANI pH sensor can be effectively applied to monitor the pH of liquids and fruits, and the measured values were close to those of the commercial pH meter.

## Author contributions

Xiangxiang Zhu: designed the experiment and wrote the draft of the manuscript. Hui Sun: conceptualization and writing – review & editing. Bin Yu: conceptualization and writing – review & editing. Lei Xu: investigation and formal analysis. Hao Xiao: investigation and formal analysis. Zhuan Fu: investigation and formal analysis. Tian Gao: investigation and formal analysis. Xiaodong Yang: investigation and formal analysis.

## Conflicts of interest

The authors declare that they have no known competing financial interests or personal relationships that could have appeared to influence the work reported in this paper.

## Supplementary Material
